# Optimizing Driving Parameters of the Jumbo Drill Efficiently with XGBoost-DRWIACO Framework: Applied to Increase the Feed Speed

**DOI:** 10.3390/s24082600

**Published:** 2024-04-18

**Authors:** Hao Guo, Lin Lin, Jinlei Wu, Yancheng Lv, Changsheng Tong

**Affiliations:** School of Mechatronics Engineering, Harbin Institute of Technology, Harbin 150001, China; ghgh8372024@163.com (H.G.); wujinlei_12@163.com (J.W.); 19b908087@stu.hit.edu.cn (Y.L.); 20b908078@stu.hit.edu.cn (C.T.)

**Keywords:** dimension reduction while iterating, ACO, driving parameter optimization, feed speed increase, jumbo drill, XGBoost, cost reduction

## Abstract

The jumbo drill is a commonly used driving equipment in tunnel engineering. One of the key decision-making issues for reducing tunnel construction costs is to optimize the main driving parameters to increase the feed speed of the jumbo drill. The optimization of the driving parameters is supposed to meet the requirements of high reliability and efficiency due to the high risk and complex working conditions in tunnel engineering. The flaws of the existing optimization algorithms for driving parameter optimization lie in the low accuracy of the evaluation functions under complex working conditions and the low efficiency of the algorithms. To address the above problems, a driving parameter optimization method based on the XGBoost-DRWIACO framework with high accuracy and efficiency is proposed. A data-driven prediction model for feed speed based on XGBoost is established as the evaluation function, which has high accuracy under complex working conditions and ensures the high reliability of the optimized results. Meanwhile, an improved ant colony algorithm based on dimension reduction while iterating strategy (DRWIACO) is proposed. DRWIACO is supposed to improve efficiency by resolving inefficient iterations of the ant colony algorithm (ACO), which is manifested as falling into local optimum, converging slowly and converging with a slight fluctuation in a certain dimension. Experimental results show that the error by the proposed framework is less than 10%, and the efficiency is increased by over 30% compared with the comparison methods, which meets the requirements of high reliability and efficiency for tunnel construction. More importantly, the construction cost is reduced by 19% compared with the actual feed speed, which improves the economic benefits.

## 1. Introduction

The drilling and blasting method is the most commonly used method for tunnel construction. Different from the shield method, which depends primarily on mechanical cutting, the tunneling footage using the drilling and blasting method is achieved by blasting the rock. The process mainly includes drilling, blasting, ventilation, support, and slag discharge. This method is more flexible in construction, lower in cost, and more adaptable to geological conditions compared with the shield method. Moreover, it is especially suitable for tunnel projects with long distances and changeable geological conditions [1]. As of 2019, the total length of the tunnels built in China is around 10,000, of which more than 90% were completed using the drilling and blasting method [2]. The whole process of the drilling and blasting method has been mechanized, while drilling is the core step. The jumbo drill, as the main equipment for the drilling process, greatly impacts on the building speed and construction quality. Further, time spent on processes such as the blasting and support of each blasting cycle is fixed, so building speed mainly depends on the feed speed of the jumbo drill. Feed speed refers to the distance that the drill bit of the hydraulic rock drill drills on the tunnel face in a unit of time when the machine performs the blasthole drilling operation. The unit is m/min. Under specific working conditions, increasing the feed speed to reduce the construction cost is one of the key decision-making issues for tunnel construction [3].

Previous research showed that the feed speed of the jumbo drill is mainly affected by geological conditions and driving parameters. The geological conditions can be characterized by surrounding rock classification [4]. The driving parameters include impact pressure, feed pressure, rotational pressure, water pressure, and water flow [5]. The influence of driving parameters on feed speed is reflected in two aspects. First, the matching relation between driving parameters will affect the feed speed. If the impact pressure and rotational pressure do not meet the performance required by feed pressure, it will lead to abnormal drilling conditions such as sticking and idling, and then it will make the feed speed abnormal and will accelerate drilling tool wear. The reasonable matching of the above driving parameters is the basis for normal drilling. Second, driving parameters are not perfectly positively correlated with feed speed. Excessive feed pressure, for example, can cause the drill pipe to bend significantly, which, in turn, decreases feed speed. Under specific working conditions, it is practical to optimize driving parameters to increase the feed speed.

**Figure 1:**
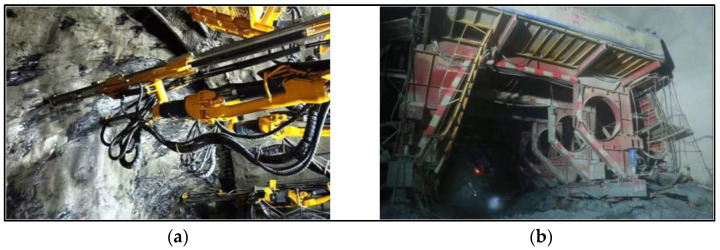
The complex working conditions of the jumbo drill: (**a**) a jumbo drill works in the complex formation; (**b**) a jumbo drill is washed away by more than 100 m due to mud and water in the rush.

Tunnel construction is of high risk. An unreasonable tunneling process can easily cause serious engineering accidents, such as face collapse, rock bursts, mud, and water in a rush [6]. For this reason, the matching relation of driving parameters and corresponding feed speed is required to ensure high reliability. Meanwhile, due to the complex working conditions, which are mainly reflected in the changeable geological conditions and the limitation of the operating status of the service equipment, driving parameters and corresponding feed speed should respond to the changes in time to avoid invalid optimized results [7]. That is to say, the optimized results need to have high efficiency. Figure 1 shows the complex working conditions and the mud and water in the rush accident.

The driving parameter optimization for the jumbo drill under specific working conditions can be represented as follows.

(1) $$\begin{array}{c} Obj=\max(FS)\\ S.\;t.\;\left\{\begin{array}{c} X_{1}=G\\ D_{l}\leq X_{2}\leq D_{u} \end{array}\right. \end{array},$$

where Obj is the objective function, and FS is the feed speed of the jumbo drill. Constraints (X) depend on the specific working conditions, including constraints of geological conditions ($X_{1}$) and constraints of driving parameters ($X_{2}$), such as feed pressure and impact pressure [8]. $D_{l}$ and $D_{u}$ are the lower bound and upper bound of the value range of the driving parameters, respectively, in the current state of the equipment.

Generally speaking, methods of driving parameter optimization in tunnel engineering include experiments, numerical simulations, and optimization algorithms. The experimental method refers to testing the influence of the driving parameters on the optimized objective using experiments to obtain the optimal solution [9]. However, the experiment environment is often difficult to build. What is more, this method is low in efficiency, so it cannot meet the requirement of a timely response to the changeable working conditions. The numerical simulation method refers to simplifying the tunnel engineering problem into a mathematical model and obtaining the approximate solution by numerical analysis [10]. The impact of driving parameters on the optimized objective can be efficiently reflected by this method, which meets the requirement of high efficiency. However, the reliability of the results is generally low because the simulation process needs to simplify the boundary conditions, and the constitutive model of the analysis software does not necessarily match the engineering problem, which may affect the results [11].

With the development of artificial intelligence and its application to engineering, optimization algorithms represented by swarm intelligence algorithms have gradually been used in tunnel engineering [12]. Mikaeil R et al. employed a fuzzy C-mean clustering algorithm to assess the risk level of tunnel construction based on geological conditions, groundwater flow and other factors [13]. Moreover, the effectiveness of the intelligent model was verified on Iranian road tunnels. Afradi A et al. used the fuzzy logic method to predict the penetration rate of the tunnel boring machine, and the input parameters included compressive strength, density of the rock, and so on [14]. The results show that the prediction accuracy is better than the traditional mechanistic models. There are two key issues for swarm intelligence algorithms: (1) establish the evaluation criteria of the optimization problem, which is also called the evaluation function; (2) establish the heuristic rules that realize rapid convergence. The main problems existing in the above issues are as follows.

I. The evaluation function reflects the mechanism of the driving parameters to the optimized objective to a certain extent. So, the accuracy is very high under specific working conditions, which ensures the high reliability of the optimized results [15]. The solution of the evaluation function is mainly based on the model-driven method. Concretely, a mathematical model was constructed according to prior knowledge, such as the principles of the engineering problem and data distribution characteristics, and experimental data were used to fit the coefficients [5]. However, the difficulty of constructing a mathematical model lies in the fact that the considerable and interrelated domain knowledge of engineering problems makes it hard to determine the main parameters and the form of the model [16]. The existing models for predicting feed speed are in the form of a polynomial with two features [17,18]. First, these models cover the relationship between a single driving parameter and feed speed without considering the influence of the matching relation of the driving parameters on feed speed. Second, these models are built based on specific experimental conditions, so the ability to generalize to other working conditions is poor. Therefore, the above models based on the model-driven method are not suitable for tunnel engineering with complex working conditions [19]. The data-driven evaluation function can handle the nonlinear relationship between the high-dimensional parameters. Moreover, the model can be continuously updated according to the data under complex working conditions to improve the applicability of the evaluation function cross-working conditions. Guo D et al. mixed random forest and LSTM algorithms to establish an intelligent driving model for the tunnel boring machine [20]. Multiple variables, such as geological conditions and torque, are used to predict the cutter speed and penetration speed, and they provide a decision-making reference for the operation process. Kim D employed an ensemble learning algorithm to predict the surface settlement of the urban tunnels according to more than 40 input parameters [21]. The accuracy of the method was improved by more than 10% compared with the traditional method and is applicable to complex geological conditions.

II. Swarm intelligence algorithms have been applied in the field of tunnel optimization. Wang H et al. proposed a differential evolution-based multi-objective genetic algorithm to optimize the feed speed and cutter speed of the tunnel boring machine under different geological conditions [22]. Moreover, the results show that the optimization results are better than those of the manual experience. Kim K et al. used the particle swarm optimization (PSO) algorithm to optimize the drainage system of the undersea tunnel and simulated the water boundary conditions of the optimal drainage system [23]. The optimized results reduce the construction cost under the premise of ensuring hydraulic stability. The shortcomings of swarm intelligence algorithms are that the convergence rate is greatly affected by initial values and the search rate is low, leading to the difficulty in meeting the requirement of high efficiency for tunnel construction [24].

Given the above problems, the XGBoost-DRWIACO framework is proposed in this paper. Moreover, the research findings are applied to driving parameter optimization to increase the feed speed of the jumbo drill. The following research is carried out.

I. The XGBoost-DRWIACO framework is established in Section 2. Part 1 of the framework is a high-accuracy prediction model for feed speed based on XGBoost. According to construction data in different working conditions, the model realizes effective mapping between driving parameters and feed speed. As an evaluation function, it is suitable for driving parameter optimization under complex working conditions. Part 2 of the framework is an improved ant colony algorithm based on dimension reduction while iterating strategy (DRWIACO). The algorithm resolves three situations that lead to the inefficient iteration of the ant colony algorithm (ACO), thereby improving efficiency compared with ACO.

II. The effectiveness of the prediction model for feed speed and the DRWIACO are verified in Section 4. Then, driving parameter optimization using the XGBoost-DRWIACO framework is carried out on the construction dataset. The optimized results with high accuracy and efficiency are proved to meet the needs for tunnel construction and improve the economic benefit.

## 2. Methodology

### 2.1. Motivation

**Figure 2:**
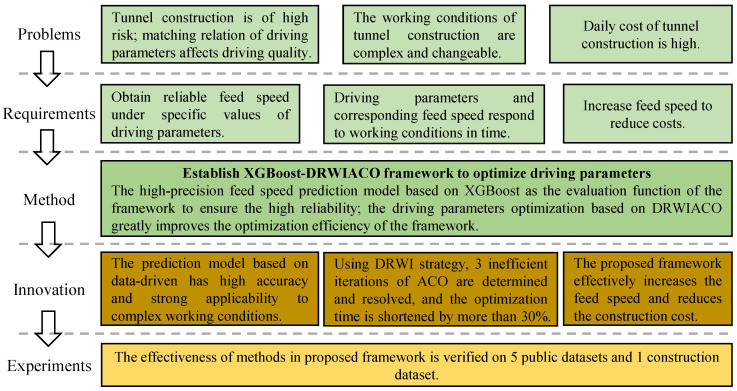
The research motivation of driving parameter optimization.

To clarify the engineering requirements, difficulties, solutions, and benefits of driving parameter optimization, the research motivation of this paper was organized as shown in Figure 2.

### 2.2. Prediction Model for Feed Speed Based on XGBoost

The evaluation function of the framework, with the high accuracy of the prediction model for feed speed, contributes to the high accuracy of the optimization algorithm, resulting in optimized results with high reliability. Moreover, the simpler the structure of the model is, the shorter the runtime is, and the higher the optimization efficiency is. In this paper, a data-driven method was adopted to establish a prediction model, which has the following advantages compared with the model-driven method. Firstly, the form of the model is supposed to adapt to the data instead of being preset, and it improves the accuracy of the model for fitting the construction data. Secondly, the model can be optimized based on the continuously collected construction data to improve the adaptability to complex working conditions. Thirdly, when dealing with new working conditions, the modeling efficiency is improved by updating the model with new construction data rather than reestablishing it with simulations or experiments [25].

Based on the performance analysis, the XGBoost algorithm was selected to establish the prediction model for feed speed. The XGBoost is a boosting method proposed by Chen Tianqi in 2014. The basic idea is to combine many Classification and Regression Trees (CARTs) based on a certain strategy to form an integrated model with high accuracy [26]. Equations (2)–(4) show the principle of the integrated strategy.

(2) $$Obj=\sum_{i=1}^{n}L(y_{i},{\hat{y}}_{i})+\sum_{k=1}^{N}\Omega (f_{k}),$$

(3) $${\hat{y}}_{i}=\sum_{k=1}^{N}{\hat{y}}_{i}^{k},$$

(4) $$\Omega (f_{k})=\gamma T+\frac{1}{2}\lambda \sum_{j=1}^{T}\omega_{j}^{2},$$

where Obj is the objective function, L is the loss function, $y_{i}$ is the actual value of the *i*-th sample. ${\hat{y}}_{i}$ is the predicted value of the *i*-th sample, ${\hat{y}}_{i}^{k}$ is the predicted value of the $k^{th}$ CART ($f_{k}$). $\Omega (f_{k})$ is the regularization term. T is the number of leaf nodes of $f_{k}$. $\omega_{j}^{2}$ is the regularized score of the *j*-th leaf nodes. γ and λ are penalty terms.

According to theoretical analysis, the advantages of applying XGBoost to establish a prediction model for feed speed are as follows.

I. The second-order Taylor expansion is applied to the loss function (L) instead of only the first derivative, which makes the loss calculation more accurate. It makes the model accurate, thereby meeting the requirements of high reliability in tunnel engineering [27].

II. The regularization term ($\Omega (f_{k})$) is added to the objective function (Obj), which can prevent the model from overfitting by reducing the variance. It improves the generalization ability of the model and makes the model suitable for complex working conditions [28].

III. Values of the features of CART are sorted in advance, and then saved to a reusable block structure, which reduces the time spent on sorting. It makes the model efficient, thereby meeting the requirements of high efficiency in tunnel engineering [29].

### 2.3. An Improved Ant Colony Optimization Algorithm with High Efficiency

After modeling an efficient and accurate evaluation function, the swarm intelligence algorithm for rapid optimization is studied in this section.

#### 2.3.1. Ant Colony Optimization Algorithm and the Defects in Efficiency

The swarm intelligence algorithms mainly include genetic algorithm (GA), particle swarm optimization (PSO), ant colony optimization (ACO), glowworm swarm optimization (GSO), etc. These algorithms can deal with the optimized objective with no direct reference direction of descent and a mass of optimized parameters. Among them, ACO is widely used in engineering, with the advantages of being insensitive to initial values, having strong global search abilities, and being easy to improve [30,31]. However, the defect of the swarm intelligence algorithms is their low optimization efficiency, which leads to the problem that the optimized results of the driving parameters cannot respond to changeable working conditions in time.

The principle of ACO is as follows. Randomly place m ants, and the destination is point Q, where the food is placed. When an ant passes through path (i,j) formed by points i and j, a certain number of pheromones is released to convey information to the population. With a short path comes a high concentration of pheromones, which contributes to a large probability of the path being chosen by other ants. Thus, a positive feedback mechanism for ants to find the shortest path to point Q is formed [32]. When all ants complete a cycle, the content of pheromones on all paths needs to be updated. The transition probability of the *k*-th ant from point i to j in the *t*-th iteration is represented in Equation (5).

(5) $$P_{k}(i,j)=\left\{\begin{array}{ll} \tau_{ij}^{\alpha }(t).\eta_{ij}^{\beta }(t)/(\sum_{s\in A_{k}}\tau_{is}^{\alpha }(t).\eta_{is}^{\beta }(t)) & s\in A_{k}\\ 0 & s\notin A_{k} \end{array}\right.,$$

where $\tau_{ij}(t)$ is the content of the pheromone on path (i,j) in the t-th iteration. $\eta_{ij}(t)$ is the distance heuristic function, with $\eta_{ij}(t)=1/d_{ij}$, and $d_{ij}$ is the distance of path (i,j). α,β are the control parameters, which adjust the importance of $\tau_{ij}(t)$ and $\eta_{ij}(t)$, respectively. $A_{k}$ is a collection of points that the ants can reach in the iteration after the *t*-th iteration.

The contradiction of ACO occurs between the global search ability and the convergence rate to the optimal solution. Generally, the greater the randomness of the search space, the stronger the possibility of finding the global optimal solution. However, too much randomness causes the low utilization of prior knowledge, such as the concentration of pheromones and path distance and results in slowing the convergence rate [33]. From the point of view of the search strategy, an important reason why ACO is inefficient lies in the insufficient use of heuristic information while iterating. The result is that multiple moves of the ants provide little gain to the optimized objective. Specifically, only the information of the current point and the next point is used to guide path search without the full use of the previous path information. This is not conducive to avoiding obstacles to find the optimal solution, and ants may easily fall into the local optimum. Three situations that lower the efficiency of ACO are discussed as follows.

**Figure 3:**
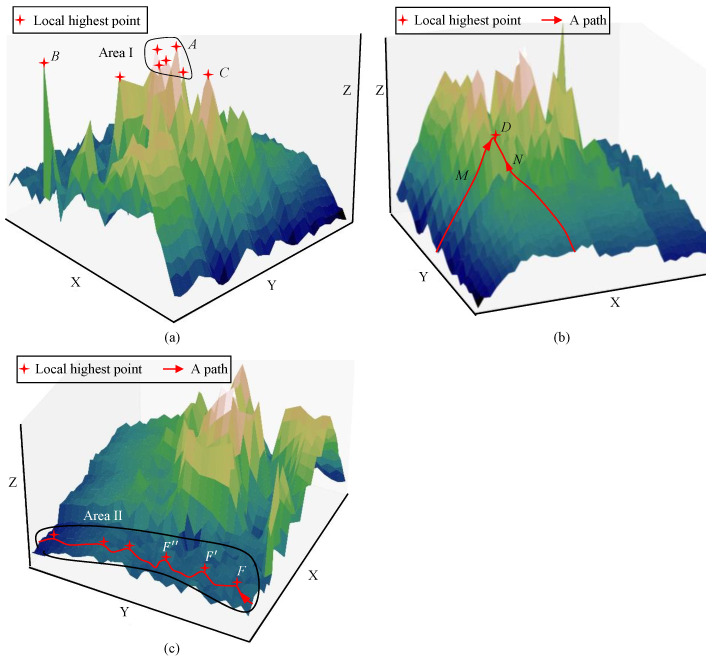
3 situations that lower the efficiency of ACO: (**a**) the ant reaches the local highest point and falls into the local optimum; (**b**) the ant converges slowly in dimension Y; (**c**) the ant converges with a slight fluctuation in dimension Y.

Situation I: Fall into the local optimum. There are several local highest points, such as points A, B, and C, in Area I. When an ant reaches point A in the *t*-th iteration, it will move to point $A^{\prime }$ with a certain transition probability in the next iteration. According to Equation (5), the greater $\tau_{AA^{\prime }}(t)$ and $\eta_{AA^{\prime }}(t)$ are, the greater the transition probability is. Hence, $A^{\prime }$ is likely to be a high point near A with high probability. In the remaining iteration, there is a high probability that the ant will move around point A in Area I, which means falling into the local optimum, as shown in Figure 3a.

Situation II: Converge slowly in a certain dimension. There are several paths for an ant to reach point D. Taking paths M and N as examples, path M relies on the step of dimension X, and path N relies on dimension Y. Obviously, the convergence rate of dimension Y is much lower than that of dimension X when the ant moves with the same step length. If the ant mainly moves in dimension Y, more steps are needed to reach point C, resulting in an inefficient iteration, as is shown in Figure 3b.

Situation III: Converge with a slight fluctuation in a certain dimension. Area II is similar to a hilly area in dimension Y. That is, there are many local highest points, but the heights of these points are not very different. When an ant moves in dimension Y, it will always move to a relatively higher point with high probability, but the gain in seeking the optimal solution is little, such as $F\to F^{\prime }\to F^{\prime \prime }$, as shown in Figure 3c. It is considered that an iteration with such slight fluctuation is invalid under a certain error.

#### 2.3.2. Dimension Reduction While Iterating Strategy to Resolve Inefficient Iterations

The main reason for the situations that lower the efficiency of ACO is the insufficient use of heuristic information. ACO only considers the heuristic information of the current point and the next point without the global movement trend of the ants. It might give rise to some ants’ inefficient movement in points or dimensions where the gain to the optimized objective is little. The dimension reduction while iterating (DRWI) strategy was proposed to make full use of information on the path that the ants have passed through in previous iterations. In the iteration after the *i*-th iteration, the following three variables of the *i*-th ant in the previous N iterations (t≥N) are considered to determine the inefficient iterations in advance, then reduce the dimension of this ant to accelerate convergence: the values of the optimized parameters ($X_{i}$), the values on the *j*-th optimized parameter of $X_{i}$ ($X_{ij}$), and the predicted values of the optimized objective ($Y_{P}^{i}$).

**Strategy to resolve Situation I.** It is determined that the ant falls into the local optimal solution if the variance of $Y_{P}^{i}$ ($D(Y_{P}^{i})$) is less than $E_{1}$ and the variance of $X_{i}$ ($D(X_{i})$) is less than $\varepsilon_{1}$, where $E_{1}$ and $\varepsilon_{1}$ are the thresholds in the previous N iterations. So, the iteration of the ant is terminated, and the optimal solution of the optimized parameter ($Best_{i}$) is the optimal solution in the previous N iterations ($\underset{X_{i}}{arc}\underset{n=1,2,\ldots ,N}{\max}(Y_{P}^{i}(n))$). The variance of multi-dimensional data is defined as follows:

(6) $$D(X_{i})=\sum_{j=1}^{J}\frac{1}{N-1}\sum_{n=1}^{N}{\left(X_{ij}^{n}-{\bar{X}}_{ij}\right)}^{2},$$

where $Y_{P}^{i}(n)$ is the value of $Y_{P}^{i}$ in the *n*-th iteration of the previous N iterations, $X_{ij}^{n}$ is the value of $X_{ij}$ in the *n*-th iteration of the previous N iterations, and ${\bar{X}}_{ij}$ is the mean of $X_{ij}$ in the previous N iterations.

**Strategy to resolve Situation II.** It is determined that the ant converges slowly in dimension j if the average rate of change of $Y_{P}^{i}$ to $X_{ij}$ ($V(Y_{P}^{i},X_{ij})$) is less than $\varepsilon_{2}$, where $\varepsilon_{2}$ is the threshold, indicating that the movement of the ant in dimension j fails to cause the change of $Y_{P}^{i}$. So, the iteration of the ant in dimension j is terminated, and the optimal solution of the optimized parameters in dimension j($Best_{ij}$) is the optimal solution in the previous N iterations ($\underset{X_{ij}}{arc}\underset{n=1,2,\ldots ,N}{\max}(Y_{P}^{i}(n))$). $V(Y_{P}^{i},X_{ij})$ is defined as follows:

(7) $$V(Y_{P}^{i},X_{ij})=\frac{1}{N-1}\sum_{n=1}^{N-1}\frac{Y_{P}^{i}(n+1)-Y_{P}^{i}(n)}{X_{ij}^{n+1}-X_{ij}^{n}}$$

**Strategy to resolve Situation III.** It is determined that the ant converges with a slight fluctuation in dimension j if the variance of the mean of $Y_{P}^{i}$ ($D({\bar{Y}}_{P}^{i})$) is less than $E_{2}$ and the variance of $D(X_{ij})$ is bigger than $\varepsilon_{3}$, where $E_{2}$ and $\varepsilon_{3}$ are the thresholds, indicating that the obvious movement of the ant in dimension j causes a fluctuant change of $Y_{P}^{i}$ in a small range. So, the iteration of the ant in dimension j is terminated, and the optimal solution of the optimized parameters in dimension j($Best_{ij}$) is the optimal solution in the previous N iterations ($\underset{X_{ij}}{arc}\underset{n=1,2,\ldots ,N}{\max}(Y_{P}^{i}(n))$). $D({\bar{Y}}_{P}^{i})$ is defined as follows:

(8) $$D({\bar{Y}}_{P}^{i})=\frac{1}{N-1}\sum_{i=1}^{N}\left({\bar{Y}}_{P}^{i}(n)-{\bar{\bar{Y}}}_{P}^{i}\right),$$

where ${\bar{Y}}_{P}^{i}(n)$ is the mean of $Y_{P}^{i}$ in the *n*-th iteration of the previous N iterations. ${\bar{\bar{Y}}}_{P}^{i}$ is the mean of ${\bar{Y}}_{P}^{i}(n)$ in the previous N iterations.

**Figure 4:**
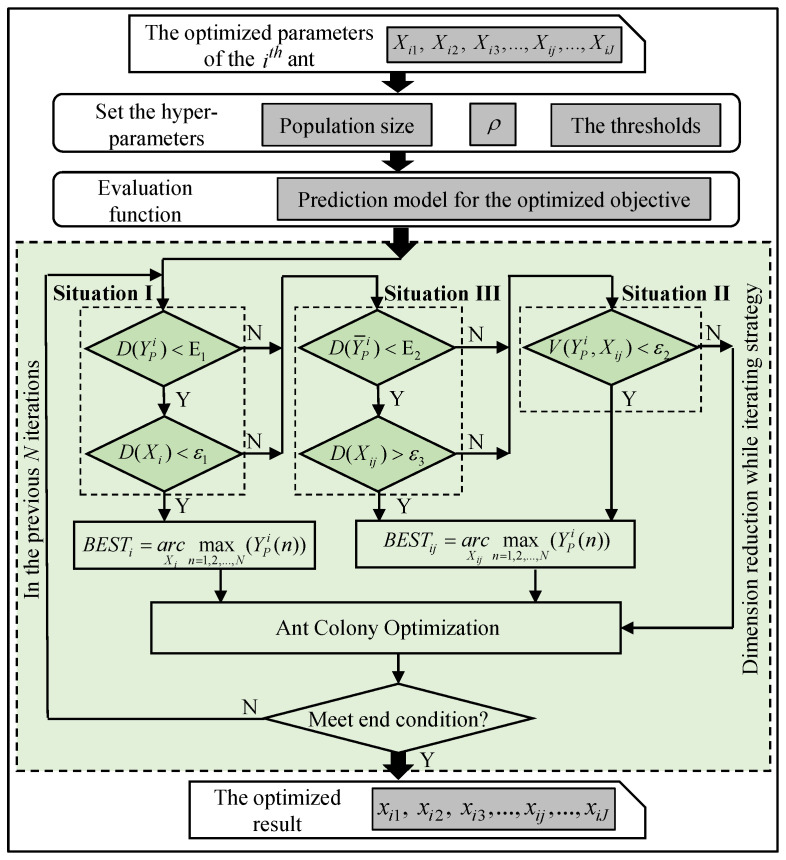
The improvement of ant colony optimization based on dimension reduction using an iterating strategy.

Figure 4 shows the improvement of ant colony optimization based on DRWI (DRWIACO). Table 1 shows the impact of the above thresholds on the process of DRWI. Generally, the stricter the threshold is set, the more difficult it is to reduce the dimension of the ants, that is, the weaker the effect of the DRWI strategy is, leading to the longer runtime of the DRWIACO.

#### 2.3.3. Evaluation Indicators of the Optimization Performance

The optimization performance of DRWIACO includes accuracy and efficiency. The accuracy is measured using the error of the optimized objective ($E_{Y}$) and the error of the optimized parameters ($E_{X}$). The efficiency is measured using the runtime (t) in the same experiment environment. Concretely, $E_{Y}$ measures the difference between the actual optimal solution of the optimized objective ($Y_{R}$) and the predicted optimal solution of the optimized objective ($Y_{P}$), as shown in Equation (9).

(9) $$E_{Y}=||Y_{R}-Y_{P}|{|}_{2}$$

**Figure 5:**
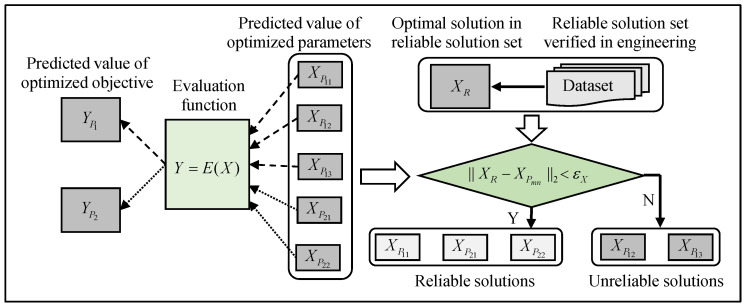
The reliability of the predicted value of the optimized parameters is judged using $\varepsilon_{X}$.

$E_{X}$ measures the difference between the actual optimal solution of the optimized parameters ($X_{R}$) and the predicted optimal solution of the optimized parameters ($X_{P}$). Since the evaluation function is normally non-monotonic, one $Y_{P}$ corresponds to several $X_{P}$. Nevertheless, the correctness of $X_{P}$ can only be checked by whether it exists in the datasets. The results of the optimized parameters that do not exist in the datasets were identified as unreliable solutions because they could not be guaranteed to be correct without verification in engineering. $\varepsilon_{X}$ was set as a threshold to judge the reliability of $X_{P}$. As shown in Figure 5, the corresponding relationship between $Y_{p_{m}}$ and $X_{p_{mn}}$ is $\left\{X_{P_{11}},X_{P12},X_{P_{13}}\}\to \{Y_{P_{1}}\right\}$ and $\left\{X_{P_{21}},X_{P_{22}}\}\to \{Y_{P_{2}}\right\}$. $X_{R}$ was obtained from the reliable solution set verified in engineering. $X_{p_{mn}}$ was considered a reliable solution if $||X_{R}-X_{P_{mn}}|{|}_{2}<\varepsilon_{X}$. To accurately measure the difference between $X_{P}$ and $X_{R}$ in each dimension, $E_{X}^{j}$, which means the difference between the actual optimal solution of the optimized parameters in dimension j ($X_{R}^{j}$) and the predicted optimal solution of the optimized parameters in dimension j ($X_{P}^{j}$), was defined as shown in Equations (10)–(12).

(10) $$E_{X}^{j}=(1+\frac{e-e_{j}}{e})e_{j},$$

(11) $$e_{j}={\left\|X_{R}^{j}-X_{P}^{j}\right\|}_{2},$$

(12) $$e=\sum_{j=1}^{J}e_{j},$$

where $e_{j}$ is the error in dimension j, and e is the sum of the errors in all dimensions.

**Figure 6:**
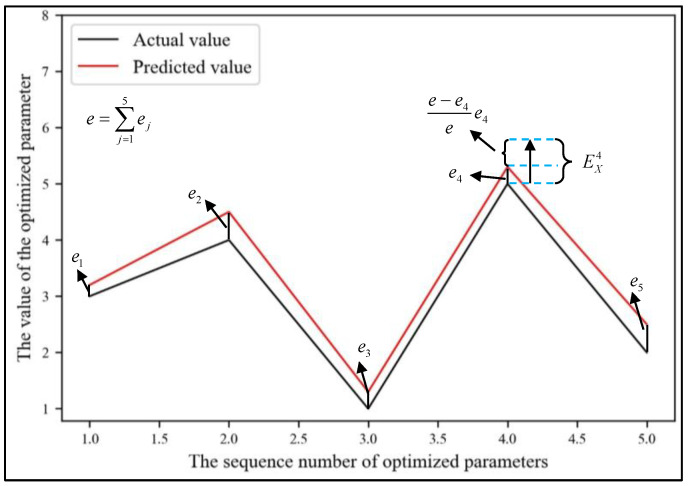
The construction process of $E_{X}^{j}$.

Equation (10) shows that $(1+\frac{e-e_{j}}{e})\geq 1$ and is determined using $\left(e-e_{j}\right)$. The bigger $\left(e-e_{j}\right)$ is, the worse the reliability of $e_{j}$ is, resulting in the enlarging of error in dimension j, as shown in Figure 6. $E_{X}$ was defined as shown in Equation (13).

(13) $$E_{X}={\left(\sum_{j=1}^{J}{\left(E_{X}^{j}\right)}^{2}\right)}^{1/2}$$

## 3. Experiment Results and Discussion

### 3.1. Data Preparation

**Figure 7:**
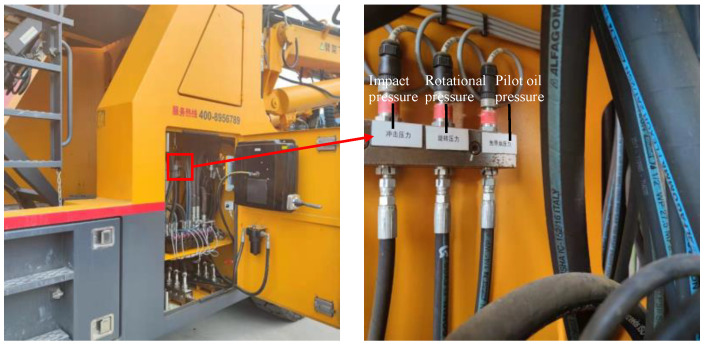
The layout of the pressure sensors and flow sensors.

The data source in this research is the construction data of the jumbo drill with three arms in the Qilinguan Tunnel Project in Hubei Province, China. It is a complex karst tunnel with a total length of 8215 m. The project started in April 2021 and has not been completed as of April 2022. Factors such as the geological properties of the rock, the type of drilling tool, and the drilling method affect the feed speed of the jumbo drill to a certain extent. However, the optimization objective is subject to a number of constraints. First of all, the objective is to adjust the driving parameters of the jumbo drill that have an influence on the feed speed so that the feed speed is as large as possible. The geological properties of the rock are the working conditions and cannot be adjusted. Secondly, the jumbo drills used in the Qilinguan Tunnel Project are unchanged, and the types of drill bits on the hydraulic rock drill are also unchangeable, with the type being B-R32-45-A63/P. The drills were manufactured by JSI ROCK TOOLs CO. LTD. in Guiyang, China. Meanwhile, due to the limitation of the geological conditions and other factors, the construction company determines a set of drilling programs. So, the drilling method basically remains unchanged. The parameters that can be adjusted within a certain range are three pressure parameters (impact pressure, feed pressure, and rotational pressure) and two borehole flushing parameters (water flow and water pressure). The three pressure parameters are controlled by adjusting the openness of the hydraulic valve, so the values of the pressure parameters are collected using the pressure sensors (MBS12003411-C1GB04) arranged here. The origin of the sensors is DANFOSS in Denmark. The values of the water flow are collected using the flow sensor (VC1F1PS/220). The origin is KRACHT in German. A piece of data is collected every 0.02 m of feed. The layout of the sensors is shown in Figure 7. The data in this research were taken from construction data in April 2021, and the amount of data was 489,823. The construction dataset was determined by screening out 458,862 pieces of data coming from the normal working state of the jumbo drill with the assistance of engineering personnel. Specifically, the normal working state is manifested as qualified drilling quality and acceptable wear of drills. The value ranges of the driving parameters are shown in Table 2.

To avoid the influence of the magnitude difference in parameters in this research, the raw data are standardized using the Z-score. The equation of a certain parameter (x) is represented in Equation (14).

(14) $$Z_{i}=\frac{x_{i}-\mu }{\sigma },$$

where $x_{i}$ is the $i^{th}$ data of x, μ is the mean of x, σ is the standard deviation of x, and $Z_{i}$ is the standardized value of $x_{i}$.

### 3.2. Performance Validation of the Feed Speed Prediction Model

#### 3.2.1. Comparison with Other Machine Learning Algorithms

To verify the performance of the predicted model for the feed speed, three regression algorithms commonly used in engineering were selected to conduct control experiments: multiple linear regression algorithm, random forest algorithm (RF), and BP neural network algorithm (BPNN). Eighty percent of the data on the construction dataset was randomly selected as the training set, and the remaining data as the test set. The experiment environment was Python 3.8, the Pentium CPU i9, 32.0 GB RAM with Windows 10. The models were obtained using 10-fold cross-validation on the training set. Ten-fold cross-validation means that the training set is randomly divided into 10 subsets. Nine subsets were selected to train the model, and the remaining one was used as the validation set to test the accuracy of the model. The process was repeated until all the subsets were used as validation sets one time so that 10 evaluation results of model accuracy were obtained. The average of all the evaluation results was taken as the accuracy of the model. Optuna was employed to determine the optimal hyper-parameters. With the help of this tool, the accuracy of the model under 10-fold cross-validation was used as an evaluation index to determine the optimal hyper-parameters of the model. The hyper-parameters are as follows. In the RF model, the number of CARTs (*M*) was 600, the learning rate (β) was 0.14, and the maximum depth of the CART was full-grown. In the XGBoost model, *M* = 600, β = 0.11, the subsample ratio was 0.86, the minimum number of leaf nodes of the CART was three, and the maximum depth of the CART tree was full-grown. In the BPNN model, β = 0.05, the number of hidden layer layers (*M*) was two, the number of hidden layer nodes (*N*) was 20, and the activation function was ReLU.

The above hyper-parameters have an important impact on the performance of the comparison models. For XGBoost, RF, and BPNN, a learning rate that is too small results in local optimum or converges slowly when training a model. The consequence is that the model is unable to adequately learn the correlation of the data, leading to the model under-fitting. Comparatively, a learning rate that is too large causes the model to oscillate or diverge during the training process. Moreover, the model fails to converge, resulting in poor model performance. M for XGBoost and RF, as well as M and N for BPNN, can change the complexity of the model, which in turn affects the performance of the model. Too small a value of the above hyper-parameters results in a model structure that is too simple to learn the data information, which, in turn, results in under-fitting. Conversely, the model with a complex structure learns the noise of the data, which, in turn, causes over-fitting.

**Figure 8:**
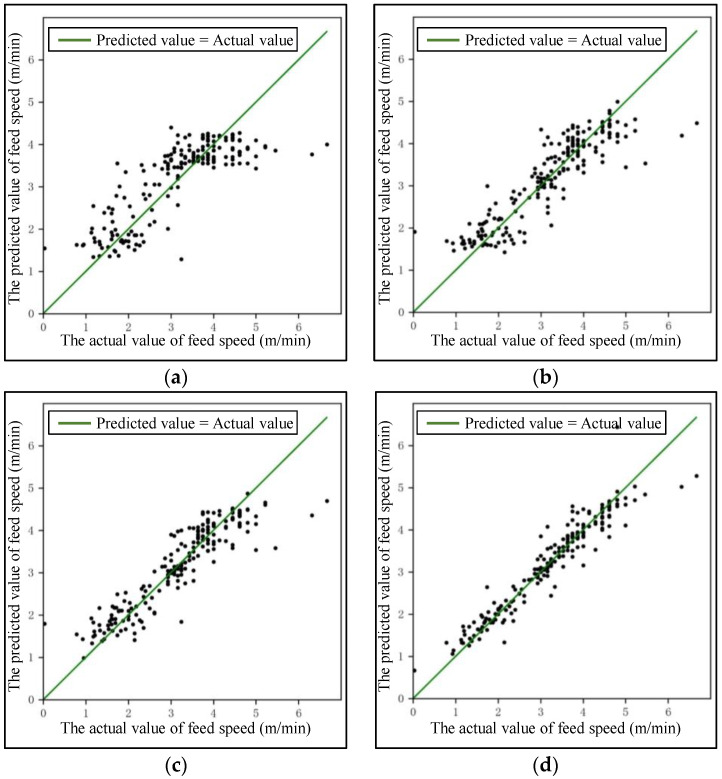
The fitting results of the prediction models based on 4 regression algorithms on the test set: (**a**) multiple linear regression model, (**b**) BPNN regression model, (**c**) random forest regression model, and (**d**) XGBoost model.

The evaluation indicators of accuracy are the coefficient of determination ($R^{2}$) and predicted error (RMSE). The efficiency was measured using runtime (t) in the same experiment environment, including runtime when training the model ($t_{\mathrm{train}}$) and runtime when testing the model ($t_{\mathrm{test}}$). $R^{2}$ represents the extent to which input parameters explain output parameters in the model, with a range of valid values being [0, 1]. A large value of $R^{2}$ means the high accuracy of the model. *RMSE* represents the error between the predicted value and actual value, which takes a value not less than 0. The optimal model was obtained using the hyper-parameter optimization process, and the model performance was evaluated on the test set. The above training and testing process was repeatedly executed 100 times, and the results of $R^{2}$, RMSE, and t were averaged, as shown in Table 3. The purpose of repeating the process 100 times to obtain the mean is to avoid the impact on model accuracy due to the chance of sample division. Figure 8 shows the fitting effect of the predicted value and the actual value of the feed speed for 200 random samples on the test set. It shows that the accuracy of the prediction model based on XGBoost is significantly higher than that of other models. $t_{\mathrm{train}}$ and $t_{\mathrm{test}}$ of XGBoost are slightly larger than that of the multiple linear regression model, and the efficiency loss is less than 6%. Compared with BPNN and RF, the efficiency improvement of XGBoost be greater than 25%, which reflects a significant efficiency advantage. The proposed model is most likely to meet the requirements of high reliability and efficiency for tunnel construction.

#### 3.2.2. Comparison with the Model-Driven Methods

Two feed speed prediction models based on the model-driven method that are widely used in engineering were selected to verify the advantages of the proposed model. Both models use feed pressure to predict feed speed. Model 1 [18] is a polynomial function, and Model 2 [17] is a piecewise polynomial function. The coefficients of the above polynomials depend on the actual working conditions and can be fitted to the training set. Table 4 shows the performance of the above models on the test set. It is clear that the accuracy of Model 1 and Model 2 is much lower than that of the proposed model, which is comparable to the linear regression model, as shown in Table 3.

### 3.3. Verification of the Efficiency Improvement of DRWIACO

#### 3.3.1. Test Method

Step I. Standardize the data of the public dataset. Train a model that uses optimized parameters to predict the optimized objective on the dataset as the evaluation function (E(X)).

Step II. Take the objective function as Obj=max(E(X)). Select the data with the maximum value of the optimized objective in the dataset as the optimal solution.

Step III. Take the value range of the optimized parameters in the dataset as the constraints and randomly determine the initial population of ants within this range.

Step IV. Apply DRWIACO and ACO to seek the optimal solution. Use $E_{Y}$, $E_{X}$, and t to evaluate the optimization performance.

The experiment environment is Python 3.8, the Pentium CPU i9, 32.0 GB RAM with Windows 10. The key hyper-parameters of DRWIACO and ACO are set with the same value to reduce the impact of irrelevant variables on performance comparison, as shown in Table 5.

#### 3.3.2. The Reference Value Range of the Thresholds for DRWIACO

**Figure 9:**
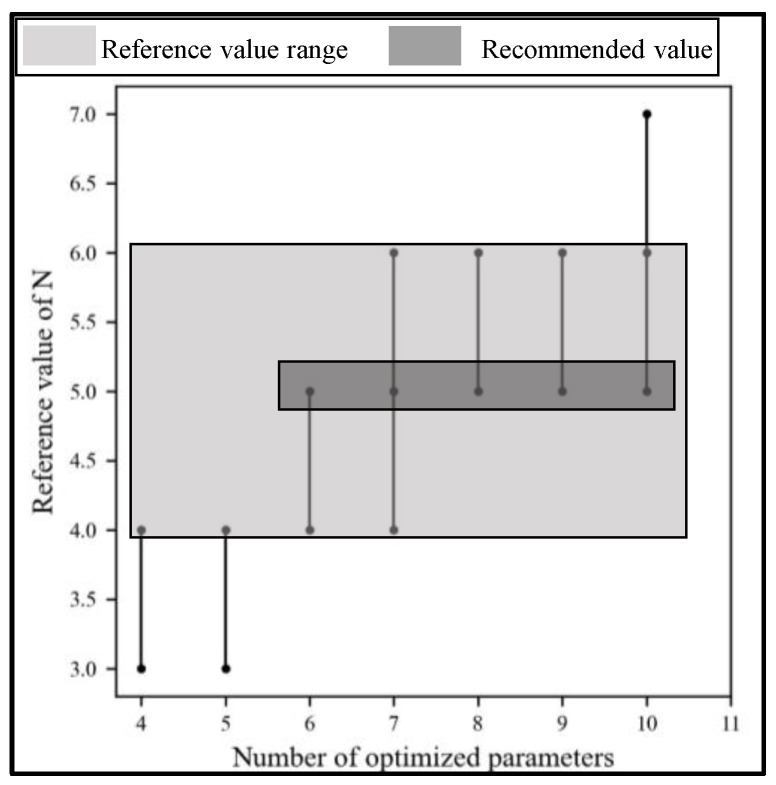
The reference value of iterative times *N* corresponding to the number of optimized parameters.

The stricter the thresholds are set, the more difficult it is to reduce the dimension, resulting in the lower efficiency of the proposed method. On the contrary, the looser the above thresholds are set, the more parameters are eliminated in the iterative process, which may lead to the optimal solution being ignored. Moreover, the accuracy of the proposed method is reduced. To synthesize the efficiency and accuracy of the method, the following evaluation indicator is set when determining the value range of the thresholds by debugging on the public datasets. Firstly, it is required that the improvement of efficiency is greater than 15% to highlight the efficiency advantages. Secondly, to ensure accuracy, it is required that the difference between the results of the optimized objective for DRWIACO and ACO is no more than 15%. The reference value range of the thresholds is shown in Table 6. Figure 9 specifically shows the reference value of iterative times *N* corresponding to the number of optimized parameters. The light gray area is the reference value range, and the dark gray area is the recommended value range with the highest selected frequency. The reference values of other thresholds are given in Appendix A. It should be noted that the above results were obtained using experiments on limited datasets. When the algorithm is applied to specific data, the values of the thresholds need to be adjusted around this value range.

#### 3.3.3. Results and Analysis

The DRWIACO and ACO were applied to five public dataset methods to seek the optimal solution. In the Computer Hardware dataset, computer hardware parameters, such as cache memory, channels, and maximum main memory, were optimized to obtain the best computer performance. In the Auto MPG dataset, parameters such as horsepower, weight, and cylinders were optimized to make the car fuel-efficient. In the Concrete Slump Test dataset, parameters such as cement, slag, and water were adjusted to minimize the concrete slump. In the Concrete Compressive Strength dataset, parameters such as water, fly ash, and superplasticizer were optimized to make concrete compressive strength maximum. In the Automobile dataset, parameters such as price, horsepower, and wheel-base were optimized to minimize the loss payment of the automobile. These datasets were chosen for three reasons. First, the selected datasets can be used to build regression models with the same type of feed speed prediction model. Second, the selected datasets have multiple features, and the effectiveness of the DRWI strategy can be highlighted. Third, they have been used in other studies for optimization performance validation, which is more convincing and reasonable. The value of $\varepsilon_{X}$ was set to 1.32, and the experiments were repeated until 10 sets of results that meet $\varepsilon_{X}$ were obtained on each dataset. The results of $E_{Y}$, $E_{X}$, and t are shown in Table 7. The runtime of DRWIACO was reduced by over 30% compared with ACO, and the efficiency improvement was more significant with the number of optimization parameters increasing. The mean of $E_{Y}$ and $E_{X}$ for DRWIACO was slightly larger than ACO, and the accuracy difference was within 5%. Meanwhile, the variance of $E_{Y}$ and $E_{X}$ for DRWIACO was slightly larger than that of ACO, which is due to the randomness of the DRWI process. Given the above, the efficiency of DRWIACO increased by more than 30%, with the accuracy loss being less than 5% compared with ACO. It proves that the DRWI strategy accelerates the convergence rate. Furthermore, the five public datasets are related to various application fields, such as construction engineering, transportation, and computer. The experimental results prove the generality of the proposed method in several engineering fields.

**Figure 10:**
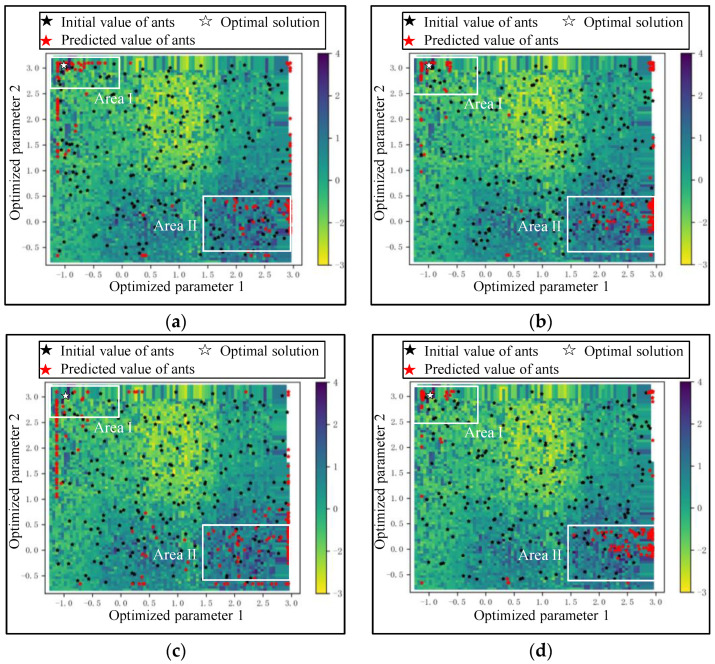
The convergence performance of DRWIACO and ACO on the Auto MPG dataset: (**a**) DRWIACO in the 100th iteration; (**b**) DRWIACO in the 200th iteration; (**c**) ACO in the 100th iteration; (**d**) ACO in the 200th iteration.

Figure 10 intuitively shows the convergence performance of DRWIACO and ACO on the Auto MPG dataset. The local optimal solutions are mainly located in Area I and II, and the global optimal solution is marked as the white five-pointed star in Area I. In Area I, many ants reach the points near the optimal solution in the 100th iteration for DRWIACO, while the optimized results for ACO are more scattered, as shown in Figure 10a,c. It indicates that the DRWI strategy is beneficial in guiding the search direction. In Area II, the optimized results for DRWIACO do not change significantly in the 100th and 200th iterations, as shown in Figure 10a,b. The distribution of ants is more scattered than that of ACO in the 200th iteration in Area II, as shown in Figure 10b,d. It reveals that the optimal solution in Area II is judged as the local optimum by the DRWI strategy, and the iterations of ants in Area II are terminated to reduce the number of invalid calculations and improve efficiency.

The CEC2017 and CEC2022 test sets are commonly used to test the performance of optimization algorithms. Four sets of tests were selected on the test sets to further validate the effectiveness of DRWIACO: I. Function F1 of CEC2017 with dimension 10; II. Function F3 of CEC2017 with dimension 30; III. Function F3 of CEC2022 with dimension 20; and IV. Function F10 of CEC2022 with dimension 20. The optimization tests were conducted using DRWIACO and ACO, respectively, and each set of tests was repeated 100 times. The results are shown in Table 8. It can be seen that the optimization accuracy of DRWIACO is slightly smaller than that of ACO in all four sets of tests, with an accuracy loss interval of [3.7%, 4.9%]. Meanwhile, the optimization efficiency improvement interval of DRWIACO is [33%, 42%]. This suggests that the conclusion that an accuracy loss of less than 5% for an efficiency improvement of more than 30% is still valid.

### 3.4. Performance Verification of the XGBoost-DRWIACO Framework

#### 3.4.1. Test Method

The driving parameter optimization to increase the feed speed of the jumbo drill under specific working conditions can be described as follows:

(15) $$\begin{array}{c} Obj=\max(M(X))\\ S.\;t.\;\left\{\begin{array}{c} X_{1}=G\\ D_{l}\leq X_{2}\leq D_{u} \end{array}\right. \end{array},$$

where M(X) is the prediction model for the feed speed, and $X=(X_{1},X_{2})$ is the input parameters of the model. $X_{1}$ is the surrounding rock classification. G is the surrounding rock classification of current working conditions. $X_{2}$ is the set of driving parameters, including impact pressure, feed pressure, rotational pressure, water pressure, and water flow. $D_{l}$ and $D_{u}$ are the lower bound and upper bound of the value range of the driving parameters, respectively, in the current state of the equipment.

The method of testing the performance of the proposed framework on the driving parameter optimization is as follows.

Step I. Set constraints, including G, $D_{l}$, and $D_{u}$. Filter out all the data that meet the constraints on the construction dataset as the reliable solution set verified in engineering ($Set_{R}$).

Step II. Select the data with the largest feed speed in $Set_{R}$ as the optimal solution.

Step III. Optimize driving parameters to maximize the feed speed using DRWIACO and obtain the predicted value of the optimal solution under the constraints in Step I.

Step IV. Employ the ACO, GA, PSO, and imperialist competition algorithm (ICA) to optimize the driving parameters as the control experiments.

The experiment environment is Python 3.8, the Pentium CPU i9, 32.0 GB RAM with Windows 10. Based on empirical equations and pre-experiments, the reasonable values of the key hyper-parameters are obtained to achieve good optimization performance for the above algorithms, as shown in Table 9.

#### 3.4.2. Model Comparisons

The above algorithms were used to optimize the feed speed under five constraints. The test was executed 20 times under each constraint. In addition to $E_{Y}$, $E_{X}$, and t, the predicted value closest to the optimal solution ($Y_{C}$) was added as the evaluation indicator to intuitively display the accuracy. Considering that the value range of the driving parameters in the construction dataset was [20,160][20,160], the raw value of $\varepsilon_{X}$ was set to 10. That is, the results of the driving parameters were considered a reliable solution when the average error of each driving parameter was within five. Table 10 shows the optimized results under two constraints. Results under all constraints are given in Appendix B. The data have been converted into the order of magnitude of raw data to display the optimized results intuitively. In terms of optimization accuracy, the mean (μ) of $E_{Y}$ and $E_{X}$ for DRWICAO was small, and the error between $Y_{C}$ and the optimal solution was controlled within 10%, indicating that the accuracy of the optimized results was high. Furthermore, the optimized results were stable since the variance ($\sigma^{2}$) of $E_{Y}$ was small. The high accuracy and stability of the optimized results met the requirements of high reliability for tunnel construction. ACO achieved the highest accuracy, which is reflected in the fact that the error between $Y_{C}$ and the optimal solution was controlled within 8%. Meanwhile, $\sigma^{2}$ was similar to that of DRWIACO, indicating a high stability of the optimized results. Compared with ACO, DRWIACO had slightly lower optimization accuracy, with an accuracy loss of less than 5%. The reason is that the DRWI strategy reduces the dimensionality of the optimization space, which prevents some feasible solutions from being accurately obtained. This reflects the strategy of sacrificing some accuracy for efficiency improvement. Compared with PSO and ICA, which have the lowest optimization accuracy, the accuracy improvement of DRWIACO was greater than 50%. Moreover, the accuracy improvement was greater than 10% compared with GA. This is due to the fact that the DRWI strategy determines three inefficient iterative situations, which makes the subsequent ants avoid the inefficient paths to improve the possibility of obtaining more feasible solutions.

In terms of optimization efficiency, compared with the ACO and GA with higher accuracy, the runtime of DRWICAO was the smallest, with t≤5 s, and the optimization efficiency was improved by more than 30%. The optimization efficiency of PSO was slightly smaller than that of DRWIACO, with an efficiency loss of about 15%. However, the optimization accuracy of PSO was poor and could not meet the requirements of high accuracy for tunnel engineering. The accuracy and efficiency of ICA are much smaller than that of DRWIACO, which may be due to the method’s poor ability to generalize engineering data. It should be noted that the runtime of DRWIACO is within 5 s, which basically meets the optimization efficiency requirements of the jumbo drill with slow building speed.

The construction data of the Qilinguan Tunnel Project in May 2022 contain more than 1000 samples, with the surrounding rock classification being III. Model comparison experiments were conducted on the above samples to test the robustness of the proposed framework under complex geological conditions. The training and testing steps of DRWIACO and the four comparison methods were the same as above. Moreover, the experimental results under Constraint 1 are shown in Table 11. It illustrates that the error between $Y_{C}$ and the optimal solution was still less than 10%. Furthermore, μ and $\sigma^{2}$ of $E_{Y}$ were small, indicating that the optimization accuracy and stability are good. Meanwhile, compared with ACO and GA, the optimization efficiency of DRWIACO was improved by 31%. This indicates that the proposed method still meets the requirements of high reliability and high efficiency under varying geological conditions; that is, the DRWIACO framework is robust.

### 3.5. Analysis of the Economic Benefits

The construction cost difference between the actual feed speed and optimized feed speed using the XGBoost-DRWIACO framework was compared in the construction data set, and the improvement of the economic benefit was verified. The construction dataset covered engineering data in 12 days. The surrounding rock classification was IV, and the excavation method was the top heading and bench excavation method. The machine worked one shift per day, and the footage for each shift was 3.8 m. According to surrounding rock conditions and construction needs, the actual blasthole depth was 4 m–5 m. The number of blastholes in the tunnel section was 180; that is, 60 blastholes were drilled for each drilling arm. The cost breakdown is shown in Table 12.

The unit output cost ($C_{e}$) of the jumbo drill was applied to measure the construction cost [34]. The formula is as follows.

(16) Ce=CLabor+CRent+CElecQF,

where $Q_{F}$ is the total drilling distance of the jumbo drill, and $t_{i}$ is the drilling time of the *i*-th shift.

**Figure 11:**
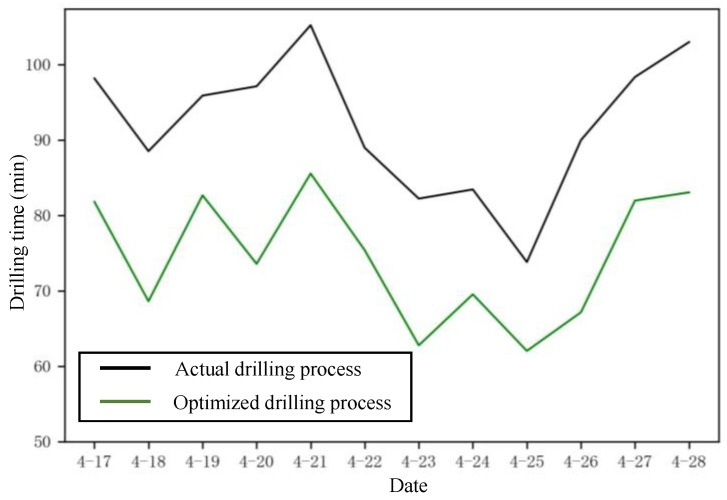
Drilling time of actual drilling process and optimized drilling process.

During the actual drilling process, the feed speed was set by the operators according to the engineering experience, which is generally 2.5–3.5 m/min. $t_{i}$ can be calculated using Equation (17). The drilling time in both modes is shown in Figure 11.

(17) $$t_{i}=\frac{D_{i}^{h}}{FS_{i}}\times N^{h},$$

where $D_{i}^{h}$ is the drilling depth of the *i*-th shift, $FS_{i}$ is the feed speed of the *i*-th shift, and $N^{h}$ is the number of blastholes for each drilling arm.

According to Equations (16) and (17), $C_{e}$ of the actual drilling process is CNY 1.5443 (USD 0.2134, EUR 0.2005) per meter, while $C_{e}$ of the optimized drilling process is CNY 1.2499 (USD 0.1727, EUR 0.1623) per meter. The construction cost was reduced by 19.06% by increasing the feed speed using the XGBoost-DRWIACO frame, which improves the economic benefit. In the U.S. or Europe, the labor cost, rent cost of the machine, electricity cost, etc., are higher than the cost in China. Therefore, it can be roughly concluded that applying the proposed feed speed optimization method of the jumbo drill to the U.S. and Europe would be more economically efficient.

## 4. Conclusions

To fulfill the requirements of high reliability and efficiency for optimizing the driving parameters of the jumbo drill, a method based on the XGBoost-DRWIACO framework to optimize the driving parameters to increase feed speed was proposed. This method solves the defects of low accuracy under complex working conditions for the model-driven evaluation function and low efficiency of swarm intelligent algorithms. The optimized results meet the above-mentioned requirements in tunnel engineering and reduce construction costs. This method has the potential to be applied to tunnel construction decision-making and production automation. The contributions of this paper are as follows.

I. An idea of using the data-driven method to establish the evaluation function was proposed, and a prediction model for feed speed based on XGBoost was established. The accuracy of the model under complex working conditions is better than that of the model-driven methods and the comparative data-driven methods.

II. DRWIACO was proposed to resolve three situations that cause the inefficient iterations of ACO. It shows that the efficiency of DRWIACO increased by more than 30%, with the accuracy loss being less than 5% compared with ACO in five public datasets.

III. The experimental results reveal that the error of DRWIACO was less than 10%, and the efficiency increased by over 30% compared with the comparison methods, which meet the requirements of high accuracy and efficiency for tunnel construction. It demonstrates a 19% increase in economic efficiency by comparing the cost before and after optimization.

It should be noted that six thresholds need to be assigned in DRWIACO. The reference value range was given by experiments in limited datasets. The values of the thresholds should be adjusted within this value range based on the specific dataset. It is necessary to further study the relationship between optimized parameters and the values of the thresholds to simplify the thresholds assignment.

## Figures and Tables

**Table 1:** The impact of the thresholds on the performance of DRWIACO.

Threshold	Tendency	The Number of Terminated Optimized Parameters	Runtime
N	+	−	+
$E_{1}$	−	−	+
$E_{2}$	−	−	+
$\varepsilon_{1}$	−	−	+
$\varepsilon_{2}$	−	−	+
$\varepsilon_{3}$	+	−	+

Remark: + indicates an upward trend, − a downward trend.

**Table 2:** The value ranges of the driving parameters on the construction dataset.

Driving Parameter	Impact Pressure (bar)	Feed Pressure (bar)	Rotational Pressure (bar)	Water Pressure (bar)	Water Flow (L/min)
Minimum	0	0	0	20	55
Maximum	190	210	130	30	120

**Table 3:** Performance of the prediction model for the feed speed based on 4 regression algorithms on the test set.

Algorithm	$R^{2}$	RMSE	$t_{\mathrm{train}}$ (s)	$t_{\mathrm{test}}$ (s)
Multiple linear regression	0.5652	0.786	2.004	0.0294
BPNN regression	0.8034	0.357	3.976	0.0466
Random forest regression	0.8527	0.261	2.977	0.0397
XGBoost regression	0.8734	0.247	2.121	0.0311

**Table 4:** Performance of the models based on model-driven method and proposed model on the test set.

Model	$R^{2}$	*RMSE*	$t_{\mathrm{train}}$ (s)
Model 1	0.5332	0.791	1.224
Model 2	0.5921	0.702	1.001
XGBoost Model	0.8651	0.251	2.155

**Table 5:** The values of key hyper-parameters for DRWIACO and ACO on the public datasets.

Hyper-Parameter	ACO	DRWIACO
Population size (m)	200	200
Volatilization rate of the pheromone (ρ)	0.3	0.3
Maximum iterations ($T_{\max}$)	200	200
Control parameter (α and β)	α=1; β=5	α=1; β=5

**Table 6:** The reference value range of the thresholds for DRWIACO on the public datasets.

Hyper-Parameter	$E_{1}$	$E_{2}$	$\varepsilon_{1}$	$\varepsilon_{2}$	$\varepsilon_{3}$	N
Reference value range	0.01–0.05	0.08–0.25	0.12–0.50	0.06–0.20	0.65–0.85	4–6

**Table 7:** Comparison of optimization performance for DRWIACO and ACO in 5 public datasets.

Dataset	Algorithm	$E_{Y}$		$E_{X}$		t (s)	
		Mean	Variance	Mean	Variance	Mean	Variance
I with 6 optimized parameters	ACO	0.364	0.176	0.852	0.255	9.05	1.431
	DRWIACO	0.387	0.185	0.896	0.273	6.74	0.962
II with 7 optimized parameters	ACO	0.401	0.463	1.034	0.565	15.36	1.674
	DRWIACO	0.426	0.527	1.167	0.621	11.33	1.024
III with 7 optimized parameters	ACO	0.454	0.505	1.125	0.581	16.87	1.854
	DRWIACO	0.465	0.529	1.236	0.614	10.94	1.145
IV with 8 optimized parameters	ACO	0.491	0.641	1.297	0.741	24.71	2.124
	DRWIACO	0.522	0.695	1.311	0.843	16.45	1.247
V with 14 optimized parameters	ACO	0.636	0.855	1.305	1.024	50.56	3.211
	DRWIACO	0.674	0.964	1.320	1.127	31.54	1.545

Remark: Names of the datasets are as follows. I. Computer Hardware; II. Auto MPG; III. Concrete Slump Test; IV. Concrete Compressive Strength; V. Automobile.

**Table 8:** Comparison of optimization performance for DRWIACO and ACO on the CEC2017 and CEC2022 test sets.

Dataset	Algorithm	$E_{Y}$		*t* (s)	
		Mean	Variance	Mean	Variance
CEC2017-F1, Dim = 10	ACO	9.62	2.33	12.58	1.74
	DRWIACO	10.09	2.64	9.46	1.13
CEC2017-F3, Dim = 30	ACO	15.66	4.02	33.72	3.79
	DRWIACO	16.42	4.66	23.74	3.01
CEC2022-F3, Dim = 20	ACO	18.25	6.37	22.60	2.54
	DRWIACO	18.93	7.65	16.34	2.04
CEC2022-F10, Dim = 20	ACO	87.32	15.97	28.78	3.50
	DRWIACO	91.59	18.44	20.41	2.97

**Table 9:** The value of the key hyper-parameter for the 5 optimization algorithms.

Hyper-Parameter	ACO	DRWIACO	GA	PSO	ICA
Population size	200	200	200	150	150
Maximum iterations	200	200	200	200	200
Addition	ρ=0.3; α=1; β=5	ρ=0.3; α=1; β=5	Crossover rate is 0.7; Selectivity is 0.5.	Inertia weight is 0.8; Learning rate is 0.35.	\

**Table 10:** The optimization performance of 5 algorithms under 2 constraints.

Constraint	Algorithm	Optimal Solution	$Y_{C}$	$E_{Y}$		$E_{X}$	t (s)
				Mean	Variance	Mean	Mean
	DRWIACO	4.70	4.91	0.604	0.286	5.914	4.23
1	PSO		4.93	1.467	0.504	16.247	5.24
	ACO		4.86	0.565	0.275	5.228	6.17
	ICA		3.84	1.751	0.600	18.014	10.87
	GA		5.46	0.516	0.257	4.501	12.74
3	DRWIACO	5.18	4.96	0.714	0.321	6.007	5.00
	PSO		4.90	1.652	0.716	19.271	5.78
	ACO		5.00	0.651	0.311	5.854	7.56
	ICA		5.90	1.574	0.722	18.523	11.71
	GA		4.53	0.627	0.307	5.252	13.17

**Table 11:** The optimization performance of 5 algorithms, with the surrounding rock classification being III.

Algorithm	Optimal Solution	$Y_{C}$	$E_{Y}$		$E_{X}$	t (s)
			Mean	Variance	Mean	Mean
DRWIACO	3.91	4.10	0.520	0.252	5.52	3.93
PSO		4.31	1.257	0.433	14.385	4.77
ACO		4.05	0.512	0.258	4.880	5.68
ICA		3.44	1.510	0.512	15.822	9.40
GA		4.20	0.450	0.242	4.143	11.00

**Table 12:** The cost breakdown for the jumbo drill with 3 arms.

Cost Item	Details	Unit Price
Labor cost ($C_{Labor}$)	Three operators	CNY 40 (USD 5.53, EUR 5.19) per person per hour
Rent cost of the machine (CRent)	One jumbo drill	CNY 400 (USD 55.3, EUR 51.9) per hour
Electricity ($C_{E\mathrm{le}c}$)	Machine power is 325 KW.	CNY 1.025 (USD 0.142, EUR 0.133) per kilowatt-hour

Source: provided by the constructors in the Qilinguan Tunnel Project.

**Figure A1:**
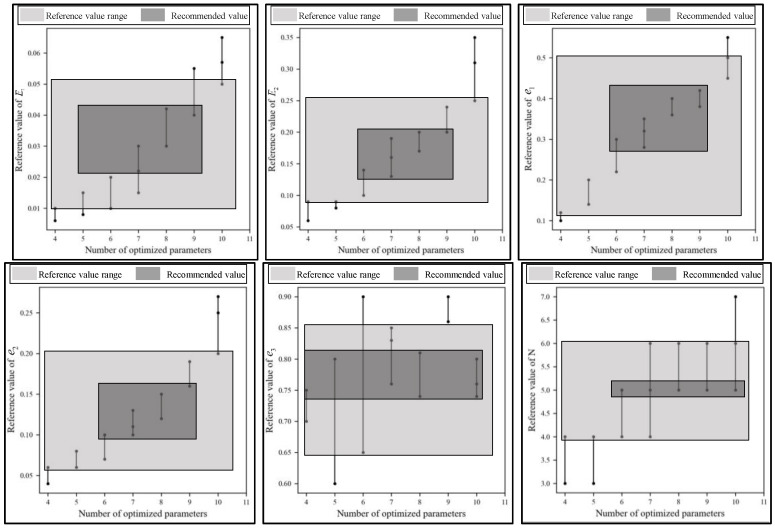
The reference value range of the thresholds for DRWIACO.

## Data Availability

The raw data supporting the conclusions of this article will be made available by the authors on request.

## Appendix B

**Table A1:** Five constraints in the construction dataset.

Constraint	Bound	Impact Pressure	Feed Pressure	Rotational Pressure	Water Pressure	Water Flow
1	$D_{l}$	90	40	45	20	55
	$D_{u}$	145	120	120	30	100
2	$D_{l}$	80	30	35	20	55
	$D_{u}$	150	120	120	30	90
3	$D_{l}$	120	45	50	20	55
	$D_{u}$	160	120	130	30	100
4	$D_{l}$	100	30	35	20	55
	$D_{u}$	120	115	125	30	95
5	$D_{l}$	80	20	30	20	55
	$D_{u}$	120	60	125	30	100

Remark: Surrounding rock classification is IV on the construction dataset.

**Table A2:** The optimization performance of 5 algorithms under 5 constraints.

Constraint	Algorithm	Optimal Solution	$Y_{C}$	$E_{Y}$		$E_{X}$	t
				Mean	Variance	Mean	Mean
1	ACO	4.70	4.91	0.565	0.275	5.228	6.17
	DRWIACO		4.93	0.604	0.286	5.914	4.23
	GA		4.86	0.516	0.257	4.501	12.74
	PSO		3.86	1.467	0.504	16.247	5.24
	ICA		5.46	1.751	0.600	18.014	10.87
2	ACO	3.91	4.09	0.485	0.260	4.904	7.54
	DRWIACO		3.69	0.514	0.272	5.467	4.65
	GA		3.75	0.443	0.254	4.252	13.86
	PSO		4.54	1.497	0.543	14.822	5.97
	ICA		4.65	1.856	0.514	14.554	9.67
3	ACO	5.18	4.96	0.651	0.311	5.854	7.56
	DRWIACO		4.90	0.714	0.321	6.007	5.01
	GA		5.00	0.627	0.307	5.252	13.17
	PSO		5.90	1.652	0.716	19.271	5.78
	ICA		4.53	1.574	0.722	18.523	11.71
4	ACO	3.14	3.24	0.394	0.154	4.574	6.45
	DRWIACO		3.10	0.416	0.161	3.613	4.21
	GA		3.04	0.347	0.124	4.274	11.97
	PSO		2.51	1.271	0.527	12.071	5.71
	ICA		2.44	1.514	0.714	10.674	9.64
5	ACO	2.62	2.75	0.341	0.124	3.348	5.97
	DRWIACO		2.76	0.382	0.158	4.562	3.94
	GA		2.51	0.310	0.117	3.107	9.37
	PSO		3.02	1.047	0.514	10.952	4.64
	ICA		3.06	1.134	0.526	12.004	8.64
